# Knockdown of the Cellular Protein LRPPRC Attenuates HIV-1 Infection

**DOI:** 10.1371/journal.pone.0040537

**Published:** 2012-07-12

**Authors:** Cameron J. Schweitzer, John M. Matthews, Christian J. Madson, Meghan R. Donnellan, Ronald L. Cerny, Michael Belshan

**Affiliations:** 1 Department of Medical Microbiology and Immunology, Creighton University School of Medicine, Omaha, Nebraska, United States of America; 2 Department of Chemistry, University of Nebraska-Lincoln, Lincoln, Nebraska, United States of America; 3 The Nebraska Center for Virology, Lincoln, Nebraska, United States of America; George Mason University, United States of America

## Abstract

HIV-1 exploits numerous host cellular pathways for productive infection. To identify novel factors involved in HIV-1 replication, HIV-1 integrase and matrix protein complexes were captured at 4 hours post infection for proteomic analysis using an affinity purification system. Leucine-rich PPR-motif containing (LRPPRC) protein, a cellular protein involved in mitochondrial function, cell metabolism, and cell-cycle progression was identified as one of the candidate HIV-1 factors. Co-immunoprecipitation RT-PCR experiments confirmed that LRPPRC associated with HIV-1 nucleic acids during the early steps of virus infection. To establish if LRPPRC was critical for HIV-1 infection, three independent LRPPRC knockdown cell lines were constructed (2.7, 3.6, and 4.1). Subcellular fractionation of these cell lines revealed differential knockdown of LRPPRC in subcellular compartments. LRPPRC was knocked down in the insoluble/cytoskeletal fractions of all three cell lines, but the 3.6 and 4.1 cells also showed a reduction in nuclear LRPPRC. Additionally, several cellular factors were downregulated and/or disrupted by loss of LRPPRC. HIV-1 infection was reduced in all three cell lines, but virus production and RNA encapsidation were unaffected, suggesting that LRPPRC was critical for the afferent stage of virus replication. Two of the three cell lines (3.6, 4.1) were refractory for murine leukemia virus infection, a virus dependent on cellular proliferation for productive infection. Consistent with this, these two cell lines exhibited reduced cellular growth with no loss of cellular viability or change in cell cycle phenotype. The early steps of virus infection were also differentially affected among the cell lines. A reduced level of preintegration complex formation was observed in all three cell lines, but viral DNA nuclear import was reduced only in the 3.6 and 4.1 cells. Combined, these data identify LRPPRC as a HIV-1 factor that is involved in HIV-1 replication through more than one mechanism.

## Introduction

Efficient replication of human immunodeficiency virus type 1 (HIV-1) requires interactions with a myriad of host cell proteins. Protein-protein interaction assays, genetic and proteomic screens have identified hundreds of candidate proteins that potentially interact with the virus during productive infection (reviewed in [1]). After entry and uncoating of its viral core, there are many critical steps during HIV-1 replication, including, but not limited to, reverse transcription of the viral RNA (vRNA) into DNA, nuclear import of the viral DNA (vDNA), and the integration of the vDNA into the host cell chromosome, transcription, specific export of unspliced viral mRNA, assembly of new virus particles, virion egress, and maturation. All of these steps involve a complex interplay between viral and cellular proteins [2], [3].

HIV-1 matrix (MA) and integrase (IN) are components of the *gag* and *pol* genes, respectively. Both proteins are incorporated into virions and present in the HIV-1 reverse transcription and preintegration complexes [4], [5], [6], [7]. The functional role of MA in virus assembly is well established. The N-terminal myristolation of MA is critical for targeting the Gag and Gag-Pol polyproteins to the plasma membrane for virus assembly [8], [9]. Although MA was among the first viral proteins identified to play a role in HIV-1 preintegration complex nuclear import, its role in the early steps of virus replication is controversial. MA is a component of both reverse transcription and preintegration complexes and contains two putative nuclear localization sequences (NLS) [10]; however, deletion of these sequences does not ablate the nuclear import process [11], [12], [13].

The principle function of IN is facilitating integration of the vDNA into the host cell chromosome. IN proteins multimerize at the ends of the newly synthesized vDNA and cleave the two proximal nucleotides at each end, resulting in a complex capable of integrating the vDNA into a heterologous target. IN contains an NLS, but similar to MA it appears to be dispensable for nuclear import of the preintegration complex [14], [15]. In the nucleus of cells, IN targets the vDNA to sites of active transcription by interacting with the chromosomal tethering protein p75/LEDGF [16]. In addition to its role in integration, IN also interacts with reverse transcriptase, is required for efficient uncoating [17], and reverse transcription [18].

Numerous cellular proteins have been identified to interact with MA and IN through in vitro assays. Yeast-two hybrid assays identified HEED [19], HO3 [20], and KIF4 [21] as MA-interacting proteins; and integrase interactor 1 [22], hRad18 [23], YY1 [24], and Gemin2 [25] as IN-interacting proteins. Other IN cofactors have been identified by their capacity to modify integration activity in vitro, including barrier to autointegration [26], high mobility group protein A2 (formerly known as HMG I(Y)) [27], and the Ku70/80 heterodimer [28], [29]. Recently, three genome-wide RNAi screens have been published to identify HIV dependency factors [30], [31], [32]. Each screen identified >200 candidate factors, but there was little overlap between them [33]. Thus far, four proteins from these screens have been validated as early HIV-1 factors: The transportin 3 nuclear import factor [34], [35] which is thought to be important for HIV-1 nuclear import, hnRNPU, a factor identified as a HIV-1 host restriction factor [36], and two cytoskeletal proteins DNAL1 and MAP4 [37].

In these studies HIV-1 IN and MA protein complexes were affinity purified from 4 h infections using a previously described in vivo biotinylation system [38]. Mass spectrometry analysis of both IN and MA complexes identified a novel candidate cellular protein, Leucine rich PPR-containing protein (LRPPRC, also known as LRP130). LRPPRC was found to interact with HIV-1 RNA and DNA during early infection. Stable depletion of LRPPRC in target cells reduced HIV-1 and MLV infection. PCR analysis of the early steps of HIV-1 infection determined that LRPPRC knockdown led to a reduction in nuclear import of vDNA and reduced PIC formation. Subcellular fractionation of the stable shLRPPRC cells revealed differential knockdown of LRPPRC in various cellular compartments. Analysis of the efferent steps of virus replication found that neither virus release nor viral RNA encapsidation were affected in cells depleted of LRPPRC. The stable knockdown of LRPPRC in 293T cells resulted in delayed cell growth compared to cells expressing a non-specific shRNA, suggesting a possible mechanism for the impact of LRPPRC expression on MLV and HIV-1 infection. These data demonstrate that LRPPRC expression is important for efficient HIV-1 replication during the afferent stage.

## Materials and Methods

### Plasmids

pNLX [39], pES [40], and pSIVmne [41] have all been previously described. The shRNA plasmids targeting LRPPRC (shLRPPRC 02, 03, 04), the non-specific (NS) shRNA plasmid, and pFlag-LRPPRC were obtained from Origene Technologies (Rockville, MD). pNLX-luc, pFB-luc and pCG-gagpol were kindly provided by Alan Engelman [42]. The plasmid standard for integration assays, pTZ19R-LTR, was constructed by inserting the HIV-1 LTR into pTZ19R (Fermentas, Glen Burnie, MD USA). The HIV-1 LTR was amplified by a standard PCR with a 5′ BamH1 and 3′ Pst1 linkers. The 5′ primer was NL1-BH1 (5′-GCGGGATCCTGGAAGGGCTAATTTGGTCC-3′; BamH1 site underlined) and 3′ primer was cNL702Pst1 (5′-GCGCTGCAGGCCGAGTCCTGCGTCGAGAG-3′; Pst1 site underlined). The PCR product was gel purified and digested with BamH1/Pst1. The digested DNA was ligated to BamH1/Pst1 digested pTZ19R. The resulting plasmid was verified by DNA sequencing (Creighton University Molecular Biology Research Core Facility).

### Cell culture and virus preparation

293T and Hela-CD4-LTR- β-gal cells (NIH AIDS Research and Reference Reagent Program, Germantown, MD) were maintained in Dulbecco's modified eagle medium (DMEM) supplemented with 10% fetal clone 3 (Hyclone, Logan, UT), 8 mM L-glutamine, 100 U/ml penicillin, and 100 µg/ml streptomycin. Hela-CD4-LTR-β-gal media was supplemented with 0.1 mg/ml G-418 and 0.1 mg/ml Hygromycin B. Viruses were produced by transient transfection of 293T cells using polyethylenimine (PEI). Briefly, cells were seeded into 10 cm dishes at 60% confluency one day prior to transfection. 15 μg of viral molecular clone (pNLX) DNA and 5 μg of vesicular stomatitis virus glycoprotein G (VSVg) expression vector pMD2.G (Addgene Plasmid Repository) were mixed with 45 μl 1mg/ml PEI in Opti-MEM (Invitrogen) minimal media. After a 10 min incubation at room temperature the PEI/DNA solution was added to each dish. HIV- luc viruses were produced by PEI transfection with 10 μg pNLX-luc and 5 μg pMD2.G. MLV-luc viruses were produced by PEI transfection with 10 μg pFB-luc, 6 μg pCG-gag-pol, and 4 μg pMD2.G. HIV-1 biotin viruses NLXIN_B_ and NLXMA_B_ were produced as previously described [38]. For all virus stock production, media was collected every 24 h for a total of 72 h, clarified by centrifugation at 4000× g for 5 min, and concentrated using Centricon-100 concentrator units as directed by the manufacturer (Millipore, Billerica MA). Viral stocks were quantified by RT activity using a previously described [^32^P]TTP incorporation assay [38], [43] and normalized amounts were used for every experiment.

### Affinity purification and mass spectrometry

The previously described BAS/BirA system was used to affinity purify protein complexes for mass spectrometry analysis [38]. Briefly, 1×10^8^ C8166-45 cells were infected by spinoculation [44] with VSVg pseudotyped HIV-1 containing biotinylated IN (NLXIN_B_) or MA (NLXMA_B_) and incubated for 4 h at 37°C. An uninfected cell lysate was prepared in parallel as a negative control. Cells were washed, lysed in RIPB buffer (50 mM Tris (pH 7.5)/ 150 mM NaCl/ 1% NP-40/0.5% sodium deoxycholate/ 0.1% SDS), clarified, and incubated overnight with streptavidin-agarose (SA) beads. The beads were washed extensively and captured proteins were eluted by boiling then separated on a large format sodium dodecyl sulfate-polyacrylamide gel electrophoresis (SDS-PAGE) system (Bio-Rad, Hercules, CA). An image of the gel was made by staining the gel with ProtoBlue Safe (National Diagnostics, Atlanta, GA), scanning, and enhancement using image software. From the image a grid was constructed and each lane was divided into 20 sections. The gel was overlaid on the grid and each section was excised and stored at −20°C. Tryptic digestion was performed using the ProteaseMAX surfactant digestion protocol (Promega, Madison WI). Following extraction the samples were purified using C-18 ZipTips (Millipore), purified samples were concentrated to ∼20 μl, and stored at −20°C until mass spectrometry analysis. Peptide samples were analyzed by the Nebraska Center for Mass Spectrometry. 10 µl of the extract solution was injected onto a trapping column (300 micron ×1 mm) in line with a 75 micron ×15cm C18 reversed phase LC column (LC- Packings). Peptides were eluted from the column using a water +0.1% formic acid (A)/95% acetonitrile: 5% water +0.1% formic acid (B) gradient with a flow rate of 270 nl/min. The gradient was developed with the following time profile: 0 min 5% B, 5 min 5% B, 35 min 35% B, 40 min 45% B, 42 min 60% B, 45 min 90% B, 48 min 90% B, 50 min 5% B. The eluting peptides were analyzed using a Q-TOF Ultima tandem mass spectrometer (Micromass/Waters) with electrospray ionization. Analyses were performed using data-dependant acquisition (DDA) with the following parameters: 1sec. survey scan (380–1900 daltons) followed by up to three 2.4 sec MS/MS acquisitions (60 to 1900 daltons). The instrument was operated at a mass resolution of 8,000. The instrument was calibrated using the fragment ion masses of doubly protonated Glu-fibrinopeptide. The peak lists of MS/MS data were generated using Distiller (Matrix Science, London, UK) using charge state recognition and de-isotoping with the other default parameters for Q-TOF data. Data base searches of the acquired MS/MS spectra were performed using Mascot (Matrix Science, v1.9.0, London, UK). The MSDB database (a comprehensive, non-identical protein sequence database maintained by the Proteomics Department at the Hammersmith Campus of Imperial College London which combines entries from TREMPL, SWISSPROT and GENBANK) (Release 02272005, 1,942,918 sequence entries) was used and the taxonomy filter was set to “human”. Search parameters used were: no restrictions on protein molecular weight or pI, enzymatic specificity was set to trypsin, and methionine oxidation was allowed as a variable peptide modification. Mass accuracy settings were 0.15 daltons for peptide mass and 0.12 daltons for fragment ion masses. Significant protein hits that matched more than one peptide with p<0.05 were identified. In the same gel fraction, protein hits matching only redundant peptides with other protein hits of higher scores were removed. Data was compiled from a total of 6 replicates and proteins unique to the infected samples were identified by comparison to the uninfected control.

### 293T shLRPPRC cells

5×10^5^ 293T cells were seeded into a single well of a six-well dish, grown overnight, then transfected with 2 μg shLRP 02, 03, 04, or a NS using TransIT-LT1 as directed by the manufacturer (Mirus Bio, Madison WI). 48 h post-transfection, the cells were seeded into four 10 cm dishes and propagated in complete DMEM media containing 1 μg/ml Puromycin (Calbiochem, La Jolla, CA) until colonies were visible. Single colonies were isolated using cloning cylinders and seeded into 96-well plates by trypsinization. Clonal cell lines were expanded and LRPPRC expression was examined by SDS-PAGE and Western blot using an anti-LRPPRC antibody (H-300; Santa Cruz Biotechnology, Santa Cruz, CA). The primary antibody was incubated overnight at 4°C, followed by HRP conjugated anti-rabbit IgG secondary antibody (GE Healthcare, Piscataway, NJ) and visualization by chemiluminescence (Pierce Biotechnology, Rockford, IL). Western blot images were acquired using an Image Station 4000R (Carestream Molecular Imaging, New Haven, CT) and, if necessary to improve quality, sharpened and adjusted for brightness/contrast using Adobe Photoshop. LRPPRC knockdown was quantified by relative optical intensity analysis using Kodak image software. The level of LRPPRC knockdown for each cell clone was calculated by comparing the level of LRPPRC expression to the level of actin, which was detected using anti-actin primary antibody (I-19; Santa Cruz Biotechnology) followed by horseradish peroxidase (HRP) conjugated anti-rabbit IgG secondary antibody. Cell lines used for assays were designated 293T -NS, -2.7, -3.6, and -4.1. Cell fractionations were carried out with the Qproteome kit according to the manufacturer's protocol (Qiagen, Valencia, CA). Western blot of fractionations were carried out as described above. The eIF4E protein was detected using antibody FL-217 (Santa Cruz), SLIRP was detected using antibody G-21(Santa Cruz), and c-Myc was detected using antibody G-4 (Santa Cruz). Detection of GAPDH was used as a loading control.

### Viral infectivity assays

For the luciferase assays, 4×10^4^ 293T shLRPPRC cells (-2.7, -3.6, -4.1, and -NS) were seeded in triplicate wells of a 24-well plate. The following day each well was inoculated with 100 µl of HIV-luc or MLV-luc and incubated for 48 h at 37°C. Cells were lysed in 100 µl M-PER solution (Pierce Biotechnology) and clarified by centrifugation at 20,000× *g* for 5 min. 25 µl of the cell lysate was loaded into a white 96-well plate and mixed with 50 µl One-glo luciferase reagent (Promega, Madison, WI). Luciferase activity was measured using a Spectramax L (Molecular Devices, Sunnyvale, CA). Uninfected cell lysate and M-PER solution only were used as negative controls for each assay. Data shown represents at least three independent experiments. For single-cycle infectivity assays, 0.25×10^6^ HeLa T4 β-gal cells were seeded into a 6-well plate and transfected the next day with 2 μg of plasmids shLRP -02, -03, -04, or a plasmid expressing a non-specific shRNA (NS) using TransIT-LT1 (Mirus Bio). 24 h post-transfection, each well of cells was trypsinized and seeded into 4 wells of a 12-well plate. 48 h post transfection, the cells in three wells were infected and assays carried out as previously described [38]. The cells in the fourth well were harvested, washed with PBS, lysed with 100 µl of M-PER solution (Pierce Biotechnology, Rockford, IL), and the level of LRPPRC knock-down monitored by Western blot as described above. The results presented represent data from nine independent infections.

### Reverse transcription and nuclear import quantification

293T shLRPPRC cells were seeded in 10 cm dishes to achieve 50–60% confluence the following day. NLX-Luc stocks were treated with 2 U/ml Turbo DNase (Ambion, Austin TX) for 1 h at 37°C. Cells were transduced with normalized amounts of NLX-Luc vector and incubated at 37°C for 24 h. Media was removed and extra-chromosomal DNA was isolated using the modified HIRT protocol [38], [45]. HIV-1 and cellular DNA was amplified using iQ SYBR Green Super Mix on an iQ5 multicolor real-time PCR detection system (Bio-Rad) using 250 nM of each primer. Late reverse transcription (LRT) viral DNA was quantified using *gag*-specific primers NL919 (5′-TTCGCAGTTAATCCTGGACTT-3′) and NL1054 (5′-GCACACAATAGAGGACTGCTATTGTA). LRT was normalized to detection of mitochondrial DNA using primers MitoFor (5′-ACCCACTCCCTCTTAGCCAATATT-3′) and MitoRev (5′-GTAGGGCTAGGCCCACCG-3′). 2-LTR circles were quantified using primers NL500 (5′-AACTAGGGAACCCACTGCTTAAG-3′) and NL9126 (5′-TCCACAGATCAAGGATATCTTGTC-3′) and normalized to the level of LRT viral DNA. Heat inactivated virus (30 min at 65°C) was used as a negative control for each experiment.

### Integration assays

Integration assays were essentially performed as described [38]. Briefly, 2×10^7^ 293T cells were infected with concentrated, VSVg-pseudotyped HIV-1 for 6 h. Cells were lysed by hypotonic swelling/dounce homogenization, the nuclei and cell debris removed by centrifugation, and the lysates snap frozen in liquid nitrogen and stored at −20°C. PIC-containing lysates were thawed on ice and treated with 20 µg/ml RNase A (Qiagen) for 10 min at room temperature prior to integration reactions. Reactions were performed in 20 mM HEPES (pH 7.4), 150 mM KCl, 1 mM MgCl_2_, 4% glycerol, 5 mM DTT. 250 µl of lysate or concentrated fraction was assayed in the presence or absence of 3 ng/µl pTZ19R target DNA (Fermentas). The reactions were incubated on ice for 5 min then transferred to 37°C for 45 min. Assays were stopped by the addition of EDTA to 8 mM. Each sample was deproteinated by the addition of SDS and Proteinase K (0.5% and 0.5 mg/ml final concentration, respectively) and incubation at 56°C for 1.5 h. DNA was extracted once with an equal volume of phenol and twice with equal volumes of phenol:chloroform:IAA. The DNA was precipitated with 0.3 M sodium acetate, 1 µl GenElute LPA (Sigma Aldrich, St. Louis, MO USA), and 2.5 volumes 100% ethanol overnight at −20°C. Samples were centrifuged at 21,000× *g* for 15 min, washed with 150 µl 70% ethanol, re-centrifuged, air-dried 5–10 min, resuspended in 20 µl Tris-EDTA, and stored at −20°C. The nested PCR strategy to measure integration activity was performed as described previously [38] except that 10-fold dilutions of pTZ19R-LTR were used as a real-time PCR standard for each real-time PCR.

### Cell cycle and proliferation assays

293T shLRPPRC cell lines were seeded at 1×10^5^ cells/10 cm plate in triplicate. At the times indicated the cells were harvested, diluted 1/500 in isotonic saline, and counted using a Z1 Coulter particle counter (Beckman Coulter, Fullerton, CA). Two independent counts were taken for each replicate per time point. For cell cycle analysis, 1×10^6^ 293T shLRPPRC cells were harvested by centrifugation, resuspended in Vindelov's reagent (10mM Tris (pH 7.6), 10 μg/ml RNase A, 75 μM propidium Iodide, and 0.1% Igepal CA-630) and incubated overnight at 4°C. Cells were analyzed at the Creighton University flow cytometry core using a FACSAria flow cytometer (BD Biosciences, San Jose, CA) and Flowjo software (Treestar Inc., Ashland, OR). MTT assays were performed on the shLRPPRC cells using the CellTiter 96 non-radioactive cell proliferation assay according to the manufacturer's specifications (Promega). A preliminary assay was performed to determine the optimum incubation time. The absorbance values were obtained using a Versamax plate reader (Molecular Devices).

### Virus release assay and viral RNA quantification

shLRPPRC 293T cells were seeded in 6-well plates at 50% confluency and transfected with pNLX using PEI as described above. Supernatants were collected at 24 h post-transfection for viral p24 ELISA and viral RNA quantification. p24 ELISAs were performed per the manufacturer's specifications (Advance Bioscience Laboratories Inc, Kensington, MD). viral RNA was isolated from the supernatant using the QIAamp Viral RNA isolation kit (Qiagen) and the relative levels quantified using iScript one-step RT-PCR kit with SYBR green (Bio-Rad, Hercules, CA) using 250 nM of each HIV-1 gag-specific primers NL919 and NL1054. PCR was performed on an iQ5 multicolor real-time PCR detection system (Bio-Rad). A dilution series of pNLX DNA plasmid was measured to generate a standard curve for the real-time RT-PCR reaction. Viral RNA encapsidation was calculated as ratio of fg viral RNA/ ng p24.

### Immunoprecipitation RT-PCR assays

For IP-RT-PCR assays of infected cells, 1×10^6^ cells were infected by spinoculation [44] with NLX + VSVg virus in the presence of 8ug/mL polybrene. After 4 h the cells were harvested, washed in PBS, and resuspended in 500 µl of IP lysis buffer (Pierce) and pre-cleared with 50 µl protein G agarose beads pre-equilibrated in lysis buffer for 1 h at 4°C. The beads were removed and the samples incubated with antibody at 1/100 dilution overnight at 4°C. The following day 50 µl of protein G agarose beads were added and incubated for an additional 4 h at 4°C. The immune complexes were washed three times for 15 min in lysis buffer and digested for 30 min at 37°C with 100 µg proteinase K in 200 µl of 50 mM Tris (pH 8.0)/1% SDS/10 mM EDTA. Viral RNA was extracted with one round each of phenol-chloroform-isoamyl alcohol (25:24:1), acid phenol-chloroform extraction, and precipitated by isopropanol with 1µl LPA. DNA was extracted once with an equal volume of phenol and twice with equal volumes of phenol:chloroform:IAA. The DNA was precipitated with 0.3 M sodium acetate, 1 µl GenElute LPA (Sigma Aldrich, St. Louis, MO USA), and 2.5 volumes 100% ethanol overnight at −20°C. RNA and DNA were purified by centrifugation for 10 min at 13,000× *g*, washed with 70% ethanol, and eluted in 50 µl Tris-EDTA. Prior to real-time PCR, the DNA was removed from all RNA samples using turbo DNA-free DNase as directed by the manufacturer (Ambion). Viral RNA and DNA were detected using primers NL919 and cNL1054.

## Results

### Identification of LRPPRC

To investigate the proteome of HIV-1 protein complexes, we utilized a previously described biotinylation system to affinity capture HIV-1 integrase (IN) and matrix (MA) protein complexes in vivo [38]. This system is based on the ability of the *E. coli* biotin ligase *BirA* to recognize and biotinylate the central lysine residue of a 20 amino acid biotin acceptor sequence (BAS). Tagged proteins can be efficiently captured with streptavidin-agarose. Two clones were constructed previously with the BAS inserted into the C-terminus of the IN and MA proteins (MA_BAS_ and IN_BAS_, respectively; [38]). Both clones efficiently produced biotinylated virus in the presence BirA. The MA_BAS_ virus was able to infect and replicate in SupT1 T cells comparable to wild-type virus, while the IN_BAS_ virus was replication defective due to a loss of integration activity.

Although defective for integration, the IN_BAS_ virus was competent for reverse transcription (data not shown). Therefore, we used both clones to capture early viral protein complexes at 4 hours post infection (hpi). High titer stocks of VSVg-pseudotyped viruses containing biotinylated IN or MA were produced by transient transfection of a 293T cell line stably expressing *BirA*. C8166-45 cells were infected by spinoculation and at 4 hpi the cells were lysed and protein complexes affinity purified with streptavidin-sepharose. For each experiment an uninfected sample was prepared in parallel as a background control. The captured protein complexes were separated by 1-D SDS-PAGE, block excised, and analyzed by liquid chromatography-tandem mass spectrometry (LC-MS/MS). An example of a Sypro stained proteomic gel is shown in Figure S1. Due to a high level of background binding to streptavidin beads, IN- and MA-associated proteins were identified by comparing the MS data from the affinity purified samples to the uninfected control samples. The top candidate cellular proteins unique to the infected samples (ranked by Mascot score and number of peptide hits) are shown in Table 1 and a list of all unique proteins identified is provided in Table S1. Four putative HIV-1 factors: eEF1A1 (EF-tu) [46], Cyclophilin A [47], [48], [49], Cyclophilin B [50], Heat shock protein 90 AA1 [51], and Heat shock protein 70 protein 9 [52], [53] were well represented in the infected samples. Although the heat shock proteins have been previously identified as an HIV-1 factor, these proteins can also non-specifically associate with sepharose-beads which are commonly used in affinity purification [54]. As, expected, there were also a large number of HIV-1 proteins identified only in the infected samples (Table S2). Both IN (Pol) and MA (Gag) were identified by MS and each was present in larger quantities within the corresponding pull-down as indicated by total peptide hits.

**Table 1:** Top candidate proteins identified in MS/MS analysis.

Gene ID	Gene name	Total (IN/MA) replicate hits	Total peptide hits	Total MASCOT score	Mean MASCOT score/replicate	Notes
1915	eEF1A1/EF-tu	20 (16/4)	51	2444	122.2	HIV-factor ^46^
10128	LRPPRC	10 (6/4)	52	2649	264.9	RNA binding
5478	Cyclophilin A	3 (2/1)	7	427	142.3	HIV-1 factor ^47–49^
5479	Cyclophilin B	3 (1/2)	3	237	79.0	HIV-1 factor ^50^
3313	HSP70 protein 9	10 (8/4)	81	4903	408.6	HIV-1 factor ^52,53^
10131	TRAP1	8 (7/1)	56	687	85.9	Mito. assoc. HSP90
3320	HSP90AA1	7 (6/1)	12	880	125.7	HIV-1 factor ^51^
3326	HSP90AB1	4 (3/1)	10	773	193.3	Constitutive form

**Figure 1:**
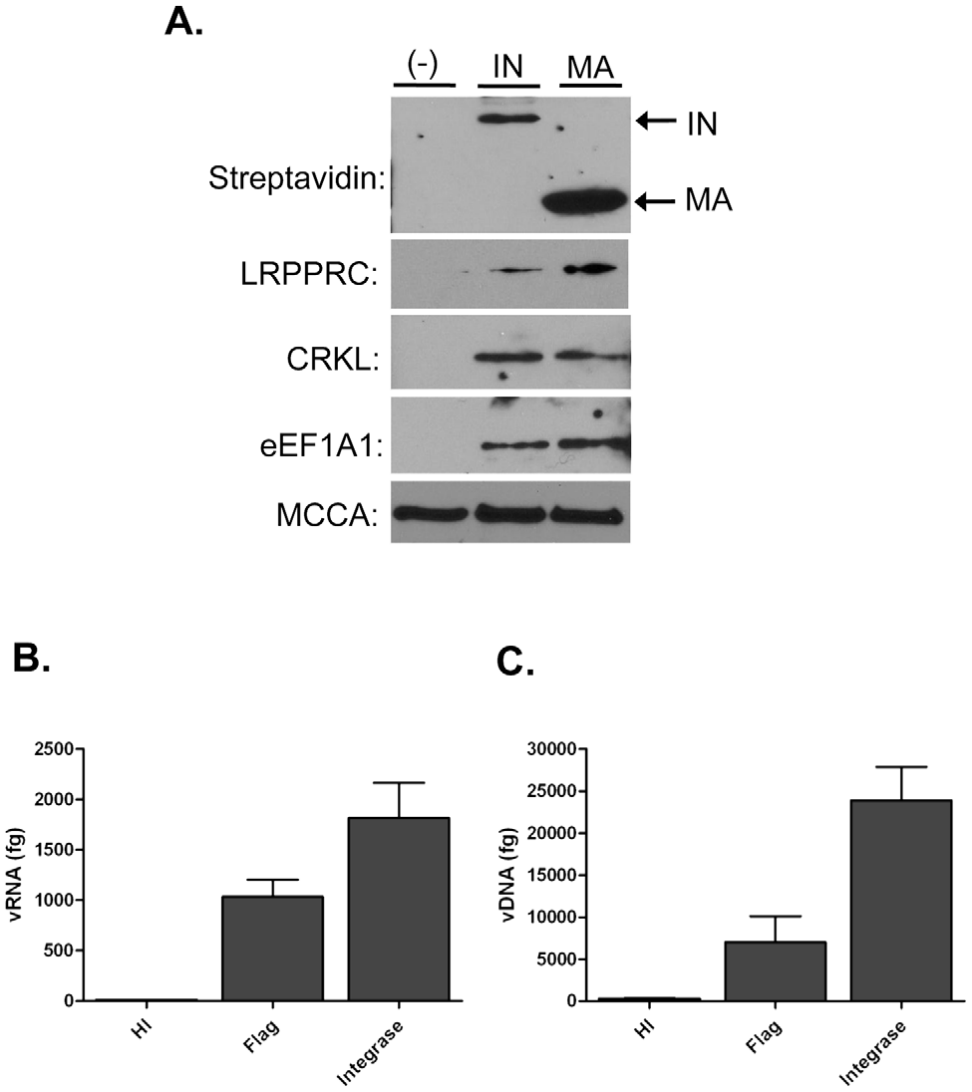
LRPPRC is present in affinity purified samples and associates with HIV-1 RNA and DNA complexes during early infection. (**A**) Western Blot analysis of affinity purified samples. C8166 cells were infected with VSVg pseudotyped NLXIN_B_ or NLXMA_B_ virus. An uninfected control was performed in parallel. Cells were lysed with IP buffer and protein complexes were affinity purified using SA-agarose. SA-agarose bound protein complexes were separated by SDS-PAGE and subjected to Western Blot with streptavidin-HRP and the indicated antibodies. (**B**) Immunoprecipitation RT-PCR in 4h infected cells. 293T cells were transfected with Flag-LRPPRC and infected with NLX. The cells were lysed, immunoprecipitated with the indicated antibodies, viral RNA isolated and quantified by real-time RT-PCR. The background level of viral RNA isolated from an isotype control immunoprecipitation was subtracted from each sample. (**C**) Immunoprecipitation PCR in 4h infected cells. Experiments were performed as in (B) except viral DNA was isolated and detected by real-time PCR. Data shown in both panels is representative of 3 replicates and error bars show SEM.

To validate the presence of the proteins identified by MS we performed Western blots on the affinity purified (AP) protein complexes (Fig. 1A). The capture of biotinylated IN and MA was confirmed using a streptavidin-HRP antibody (Fig. 1A, top panel). The protein identified with both the highest Mascot score and number of total peptide hits, Leucine rich PPR-motif containing protein (LRPPRC, also known as LRP130), was detected prominently in both the IN and MA AP samples (Fig. 1A, second panel); however, over-exposure produced a small band in the uninfected sample (data not shown). Additionally, we validated the presence of two other unique proteins in the IN and MA AP samples, CRKL and eEF1A1 (Fig. 1A, third and fourth panels). Finally, as a control we assayed for a protein ubiquitously detected in our MS analysis, MCCA, that binds endogenous biotin for metabolic processes. As expected, MCCA was detected in all samples (Fig. 1A, bottom panel). Overall these data confirm several of the MS hits and demonstrate the efficacy of the approach.

LRPPRC was discovered originally as a highly over-expressed transcript in human hepatocellular carcinoma (HepG2) cells [55], and has subsequently been described to have several diverse cellular functions. It is a member of a family of proteins that contain numerous copies of a unique structural motif called the pentatricopeptide repeat (PPR) [56]. It was characterized as a RNA/DNA binding protein found in the cytoplasm, mitochondria, and the nucleus of cells [57], [58], [59]. In addition, LRPPRC was identified as a component of the peroxisome proliferator-activated receptor coactivator 1-α (PGC-1 α) complex, which regulates energy metabolism in many tissues [60]. LRPPRC is also implicated in the human disease known as Leigh syndrome, a French-Canadian type cytochrome c oxidase deficiency [61], [62], which suggest an important role in mitochondrial metabolism. Recent work demonstrated that LRPPRC is a binding partner of eukaryotic initiation factor 4E (eIF4E), a cap-binding protein that also regulates the export of mRNAs related to cell cycle, including c-myc, Pim-1, and Cyclin D1 [63]. Additionally, LRPPRC was identified as a strong binding partner of the SRA stem-loop interacting RNA-binding protein (SLIRP) [64]. Interestingly, knockdown of LRPPRC led to a dramatic decrease in SLIRP levels and conversely knockdown of SLIRP led to a decrease in LRPPRC levels suggesting interdependent functions [64], [65].

To confirm the presence of LRPPRC in RTCs/PICs at 4 hpi and investigate its mechanism of association with HIV-1 NPCs, we performed co-immunoprecipitation (IP) assays with IN and MA, IP-RT-PCR assays for RNA interaction, and IP-PCR assays for DNA binding. 293T cells were transfected with Flag-LRPPRC and 24 hours later infected with VSVg-pseudotyped HIV-1. Four hours post-infection the cells were lysed and immunoprecipitated with isotype control, anti-Flag, or anti-integrase antibodies. LRPPRC was not found to directly co-IP with either IN or MA in replicate assays (data not shown). To determine whether LRPPRC interacted with viral RNA or DNA, immunoprecipitates were digested with proteinase K, RNA or DNA extracted, and viral copies quantified by real-time RT-PCR or PCR using *gag*-specific primers. The mouse isotype control antibody was used to measure background in each experiment and the average background binding was subtracted from each IP sample after qPCR. Infection with heat-inactivated virus was used as a negative control and anti-IN antibody was used as a positive control for these studies [66]. Immunoprecipitation of Flag-LRPPRC specifically captured HIV-1 viral RNA (Fig. 1A) and viral DNA (Fig. 1B) in early HIV-1 infection. This suggested that LRPPRC interacted with RTCs and PICs during early infection via an association with viral nucleic acids.

### Cell compartment specific depletion of LRPPRC in 293T cells

**Figure 2:**
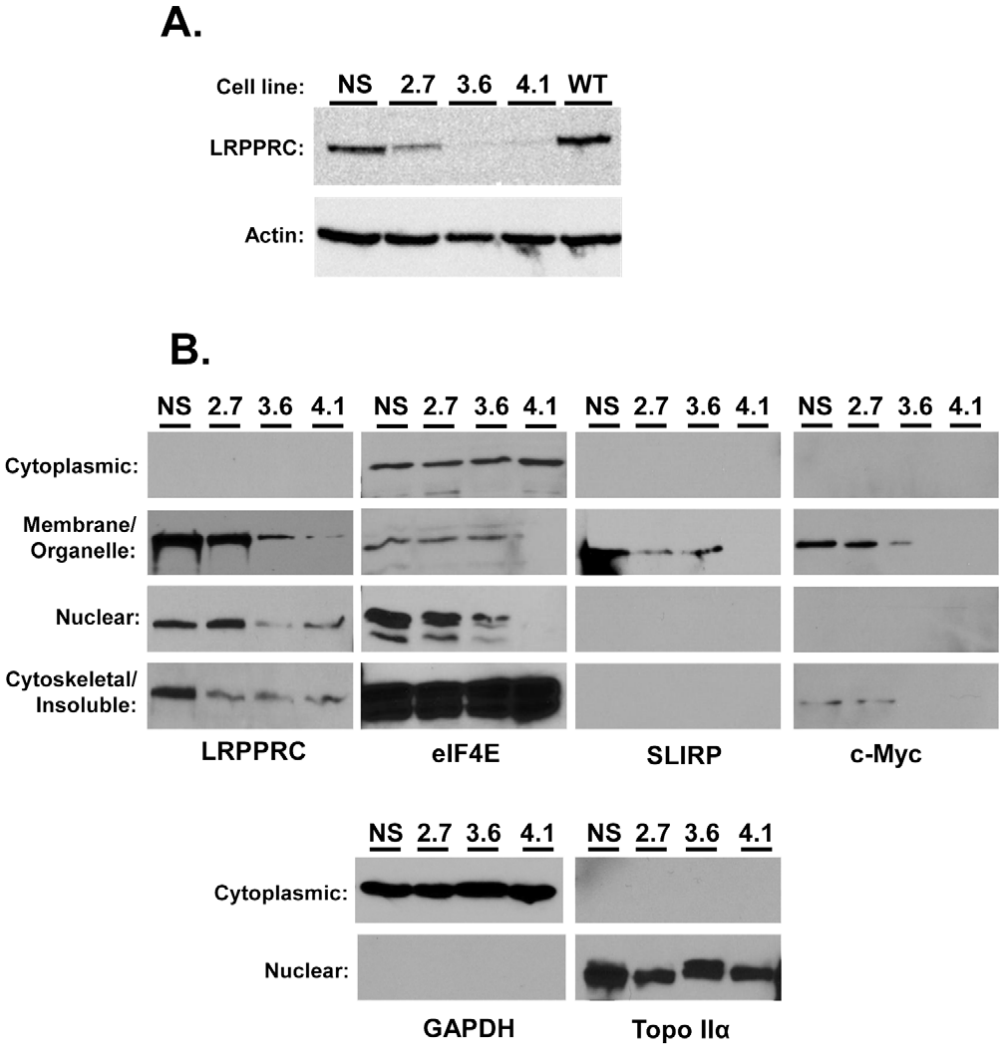
Stable knockdown of LRPPRC alters the subcellular localization of several proteins. (**A**) Level of LRPPRC knockdown in 293T cells stably transfected with the indicated LRPPRC-specific shRNAs. Knockdown was determined by comparing LRPPRC expression (top panel) to actin expression (bottom panel). WT shows the parental 293T cell line. (**B**) Subcellular fractionation of the shLRPPRC cell lines. The indicated cell lines were fractionated into cytoplasmic, membrane/organelle, nuclear, and insoluble/cytoskeletal fractions and LRPPRC, eIF4E, SLIRP, and c-Myc expression were determined by Western blot. GAPDH and Topoisomerase IIα (lower panel) were detected to ensure proper cell fractionation. Data is representative of 3 independent experiments.

To investigate the requirement of LRPPRC for HIV-1 infection, we constructed 293T cell lines stably depleted of LRPPRC by short hairpin RNA (shRNA) interference. The cells were transfected with three independent LRPPRC-specific shRNA expression constructs and selected by incubation with puromycin. Clonal cell lines were isolated by limiting dilution and screened for LRPPRC knock-down by Western blot analysis (data not shown). A cell line stably transfected with a nonspecific (NS) shRNA expression plasmid was made in parallel for control studies (293T.NS). Several cell clones were identified for each shRNA with substantial reductions in LRPPRC expression compared to the NS cells or parental 293T cell line. The cell lines that exhibited the lowest levels of LRPPRC expression for each shRNA compared to the NS stable cell line were designated 2.7, 3.6, and 4.1 and used in further experiments. Densitometry analysis of Western blots using actin as a normalization control indicated that compared to the NS control cell line, the 2.7 cells had an approximate 64% reduction in LRPPRC expression and a 99%, and 98% reduction of LRPPRC in cell lines 3.6 and 4.1 cell lines respectively (Fig. 2A).

LRPPRC is a multifunctional protein with putative roles in both mitochondrial and nuclear RNA metabolism. To characterize the knockdown of LRPPRC in each cell line we assessed LRPPRC expression in subcellular fractions. Cells were divided into cytoplasmic, membrane/organelle, nuclear, and insoluble/cytoskeletal fractions using detergent fractionation and examined by Western blot. The quality of fractionation for each sample was monitored in each experiment by the detection of GAPDH (cytoplasmic) and topoisomerase IIα (nuclear; Fig. 2B, bottom panels). No LRPPRC was detected in the cytoplasmic/soluble fraction of cells. The majority of LRPPRC protein was detected in the membrane/organelle fraction consistent with its association with mitochondria. There was only a small, if any decrease in protein expression in this fraction of the 2.7 cell line (Fig. 2B first column), while the 3.6 and 4.1 cell lines demonstrated a greater decrease. Both the 3.6 and 4.1 cell lines had a decrease in nuclear-associated LRPPRC, but the 2.7 cell line surprisingly showed no depletion in this compartment. Compared to the NS cell line all three of the shLRPPRC cell lines exhibited a strong reduction in LRPPRC expression in the cytoskeletal/insoluble fraction. Combined, these data suggest that the incomplete knockdown of LRPPRC in the 2.7 cell line was due to a lack of substantial knockdown of LRPPRC expression in the mitochondria and nuclei of those cells.

Next the expression of several cellular factors known to interact with LRPPRC was investigated. LRPPRC interacts with eukaryotic initiation factor 4E (eIF4E), an RNA regulatory protein that regulates the expression of several proteins involved in cellular proliferation. It was previously reported that knockdown of LRPPRC reduces nuclear protein levels of eIF4E which reduced the expression of several factors associated with cell cycle progression. To determine if LRPPRC knockdown altered the expression of cell cycle factors we examined their expression in the subcellular fractions of the shLRPPRC cells (Fig. 2B). eIF4E was expressed in all cell compartments in the NS cells. However there was a decrease in nuclear-associated eIF4E in the 3.6 and 4.1 cell lines (Fig. 2B, second column), which is in line with previous results indicating that knockdown of LRPPRC inhibits eIF4E translocation into the nucleus [63]. Interestingly, the 2.7 cells which have normal levels of nuclear-associated LRPPRC also exhibited normal localization of eIF4E in the nuclear fraction. Consistent with previous results, SLIRP was detected in only the mitochondrial containing fraction in NS cells, and protein levels were reduced in the 2.7 and 3.6 cell lines, and it was absent in the 4.1 cells (Fig. 2B, third column). Cellular c-Myc was also detected in NS cells in the membrane/organelle fraction with lower levels detected in the cytoskeletal/insoluble fraction (Fig. 2B, fourth column). The levels were not altered in the 2.7 cell line, but were reduced in the membrane/organelle fraction of 3.6 cells and absent in the 4.1 cell line. Combined, these data demonstrate that LRPPRC knockdown altered the expression of other cell cycle factors. Moreover, the 2.7 cell line had a different knockdown profile compared to 3.6 and 4.1 cell lines. The 2.7 cell line had a milder knockdown of LRPPRC compared to the other two cell lines and no change in the levels of eIF4E and c-Myc compared to the NS cells.

### LRPPRC knockdown decreases HIV-1 infection

**Figure 3:**
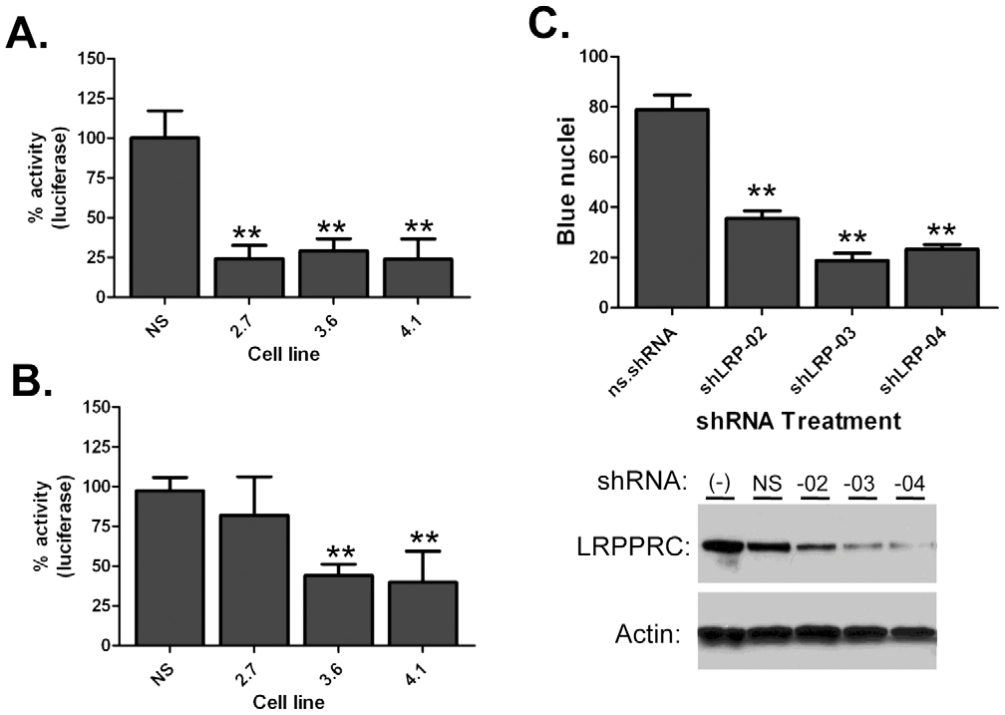
Knockdown of LRPPRC impairs HIV-1 infection. HIV-1 (**A**) and MLV (**B**) infection of cells stably depleted of LRPPRC. The indicated shLRPPRC cell lines were transduced with normalized levels of VSVg-pseudotyped HIV-1 and MLV luciferase viruses for 48 h. Data is normalized to the NS cells. ** Denotes p<0.001 as determined by two-tailed t-test, the error bars denote standard deviation, and the data is representative of at least three independent experiments. (**C**) Single cycle infectivity assays with LRPPRC-depleted cells. LRPPRC was transiently depleted in Hela-CD4-LTR-β-gal indicator cells with three LRPPRC-specific shRNAs (indicated on x-axis) 24 hrs prior to infection with HIV-1. Infectivity was measured by counting blue nuclei (indicated on y-axis). Example of transient knockdown of LRPPRC in Hela-CD4-LTR-β-gal cells is shown below the graph by anti-LRPPRC and anti-actin Western blots. Data is the average of 9 independent infections and ** denotes p<0.001 as determined by two-tailed t-test. Error bars denote SEM.

To test if LRPPRC was necessary for HIV-1 infection we measured the transduction of a HIV-1 virus engineered to express luciferase (HIV-Luc) into the LRPPRC-depleted cell lines. In each experiment the cell lines were seeded in triplicate and inoculated with VSVg-pseudotyped HIV-Luc. Virus transduction was measured by luciferase expression at 48 hours post transduction (Fig. 3A). Compared to the 293T.NS cells, an approximately 75% decrease in HIV-Luc transduction was observed for all three of the shLRPPRC cell lines, indicating that LRPPRC expression was critical for efficient infection. To determine whether the effect of LRPPRC knockdown was specific to HIV-1, the efficiency of transduction of a murine leukemia virus vector (MLV-Luc) into each cell line was measured (Fig. 3B). Interestingly, the 2.7 cell line, which has wild-type levels of nuclear LRPPRC and normal expression of eIF4E and SLIRP, facilitated MLV transduction at a comparable level to the NS cells. In contrast, a 50% decrease in MLV transduction was observed in the 3.6 and 4.1 cell lines, which have depleted nuclear and cytoskeletal/insoluble LRPPRC and reduced SLIRP and nuclear eIF4E. These data indicated that there were possibly two mechanisms through which LRPPRC depletion affected HIV-1 infectivity, and that nuclear LRPPRC was required for retroviral infection.

To corroborate the requirement of LRPPRC expression for HIV-1 infection, the effect of knockdown was investigated in a cell line that facilitates CD4-dependent HIV-1 entry. Single-round infectivity assays were performed using HIV-1 indicator HeLa-CD4-LTR-β-gal cells transiently depleted of LRPPRC (Fig. 3C). Similar to the stable cells, shRNAs 03 and 04 suppressed LRPPRC expression greater than 02 in replicate experiments. Despite this, all three LRPPRC-specific shRNA or the nonspecific shRNA were individually transfected into HeLa-CD4-LTR-β-gal cells 24 h prior to inoculation with normalized levels of HIV-1 overnight. Similar to the transduction experiments in the shLRPPRC cell lines, all three of the shRNAs to LRPPRC significantly decreased HIV-1 infectivity by at least three-fold compared to the cells transfected with the NS control plasmid. These data confirmed that LRPPRC expression was required for efficient HIV-1 infection via its natural route of entry.

### Loss of LRPPRC reduces HIV-1 PIC formation and nuclear import

**Figure 4:**
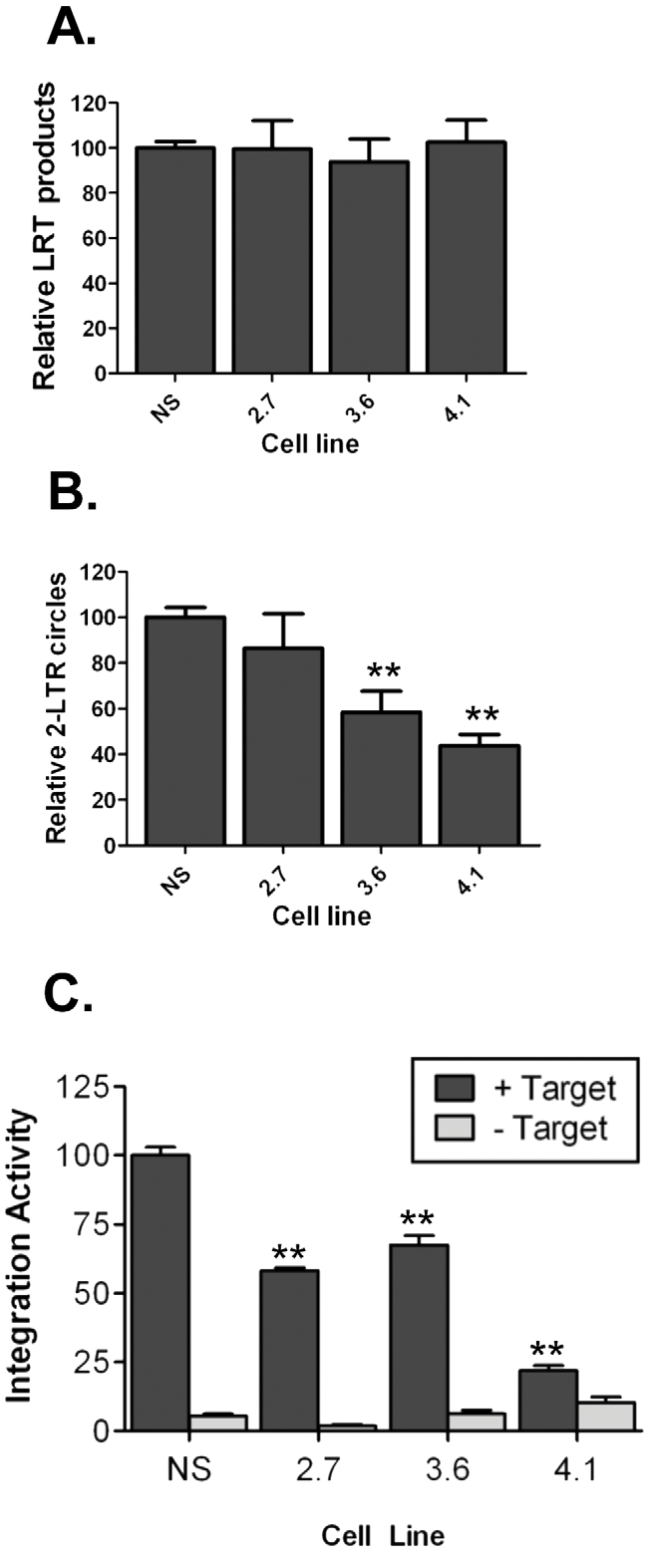
LRPPRC depletion reduces HIV-1 nuclear import and PIC formation. (**A**) LRT product synthesis in LRPPRC depleted cells. HIV-1 LRT products were measured at 24 hpi by real-time PCR and normalized to the level of mitochondrial DNA in each sample. (**B**) 2-LTR circle accumulation in LRPPRC cells. 2-LTR circles were quantified by real-time PCR and normalized to the level of late RT product in each sample. Data in panels (A) and (B) are from 3 independent experiments and the error bars denote SEM. ** denotes p<0.01 calculated by two-tailed t-test. (**C**) PIC activity in shLRPPRC infected cells. In vitro integration activity +/− target DNA was determined for each cytosolic lysate at 6 hpi. Data was normalized to the activity recovered from the NS cells and data combined from three independent experiments. The error bars denote SEM and ** denotes p<0.01 calculated by two-tailed t-test.

To identify which aspect of infection was impaired in the LRPPRC-depleted cells, we assessed reverse transcription, nuclear import, and PIC formation in the LRPPRC depleted cell lines. Late reverse transcription (LRT) and 2-LTR circle viral DNA products were measured by quantitative real-time PCR to analyze reverse transcription and nuclear import, respectively. PIC formation was quantified by in vitro integration assays. To measure viral DNA species each shLRPPRC cell line was transduced with equivalent amounts of DNase treated HIV-Luc virus and extra-chromosomal DNA was isolated at 24 hpi for qPCR analysis. To control for the extraction of DNA in each experiment the LRT product levels were normalized to the level of mitochondrial DNA in each sample. There was no difference in LRT viral DNA accumulation between any shLRPPRC cells compared to the NS cells (Fig. 4A), indicating that the depletion of LRPPRC did not affect the steps of virus replication through reverse transcription. Next, the level of viral DNA nuclear import was quantified in each cell line by measuring the accumulation of 2-LTR circles (Fig. 4B). In these experiments, the levels of 2-LTR circles were normalized to the level of LRT product in each sample to control for infection levels. There was no significant decrease of 2-LTR circle DNA in infected 2.7 cells compared to the NS shRNA-containing cells, indicating there was no defect in nuclear import in those cells. In contrast, an approximately 2-fold decrease of 2-LTR circle DNA was recovered from both the 3.6 and 4.1 cell lines, suggesting a defect in nuclear import in those cell lines.

To assess preintegration complex (PIC) formation in the LRPPRC depleted cells, each shLRPPRC cell line was transduced with equivalent amounts of NLX +VSVg and PICs harvested at 6 hpi. Lysates were normalized for viral DNA content and the level of specific integration activity measured by an in vitro integration assay. All shLRPPRC cell lines produced lower levels of PIC activity compared to NS control cells (Fig. 4C). The level of PIC activity in the 2.7 and 3.6 cell lines was approximately 2-fold less than the NS cells, and there was a >4-fold reduction in PIC activity recovered from the 4.1 cell line. These results further demonstrated that LRPPRC depletion affected the early events of HIV-1 infection.

### LRPPRC is not required for virus assembly and release

**Figure 5:**
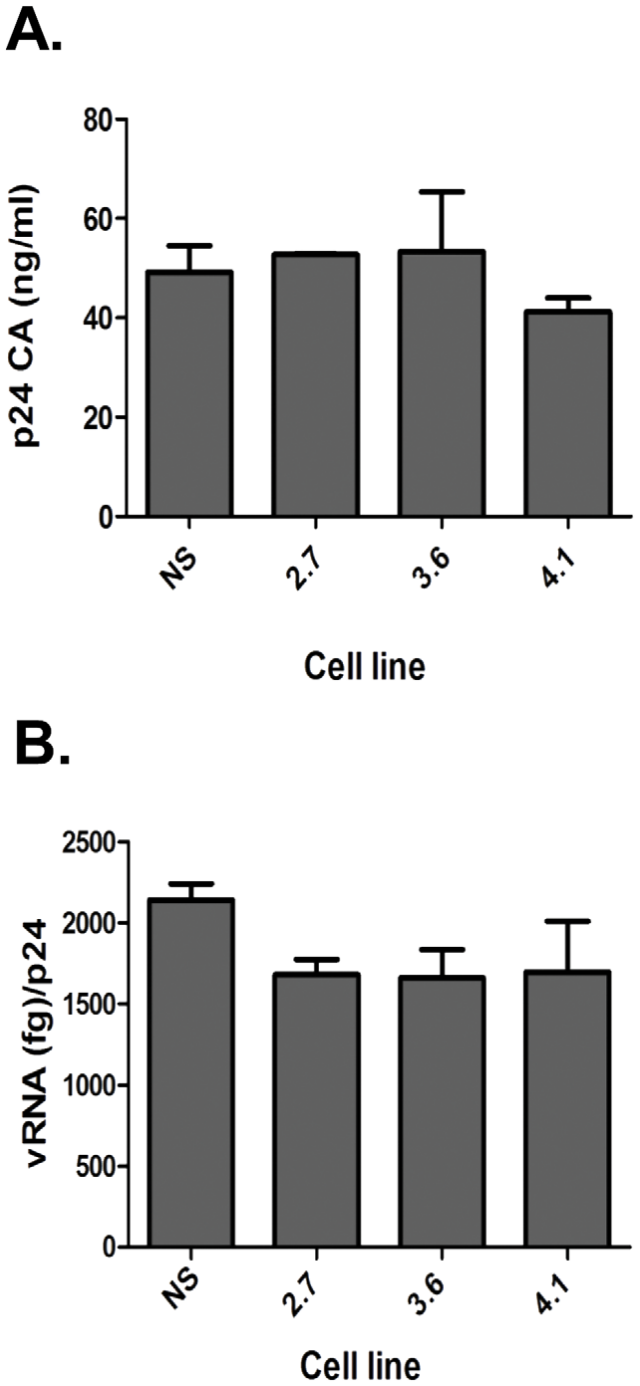
Knockdown of LRPPRC does not affect viral RNA encapsidation or virus release. (**A**) Viral release from shLRPPRC cells transiently transfected with pNLX was measured by p24 antigen ELISA. (**B**) RNA encapsidation in virus produced from shLRPPRC cells. RNA was isolated from supernatants and quantified by real-time RT-PCR. Encapsidation was calculated as a ratio of viral RNA (fg) to p24 (ng). Each sample was measured in triplicate and error bars denote SD. Data is representative of 3 independent experiments.

Thus far, the data indicated that LRPPRC was important for the early steps of HIV-1 replication. However, LRPPRC plays a role transcriptional regulation [58] and the nuclear export of mRNA [63]. To investigate whether the expression of LRPPRC affected the efferent stages of virus replication, we measured virus release and RNA encapsidation from shLRPPRC cell lines transfected with HIV-1 NLX molecular clone. Supernatants were collected at 48 h post transfection and virus production measured by p24 ELISA. In multiple experiments all three of the LRPPRC depleted cell lines produced levels of virus comparable to the NS cells (Fig. 5A), indicating there was no defect in virus expression, assembly, or release. To evaluate if LRPPRC depletion affected viral RNA encapsidation, the amount of viral RNA in supernatants was quantified by real-time RT-PCR, and the amount per ng p24 calculated (Fig. 5B). No substantial change in the levels of RNA encapsidation was observed between the NS and the LRPPRC knockdown cell lines. Moreover, the infectivity of viruses produced from shLRPPRC cells was not reduced compared to virus produced from the NS cells (data not shown) and we did not detect LRPPRC in purified virions (data not shown). Combined, this data indicated that LRPPRC was not critical for the efferent steps of virus replication.

### Growth characteristics and expression profiles of the shLRPPRC cell lines

**Figure 6:**
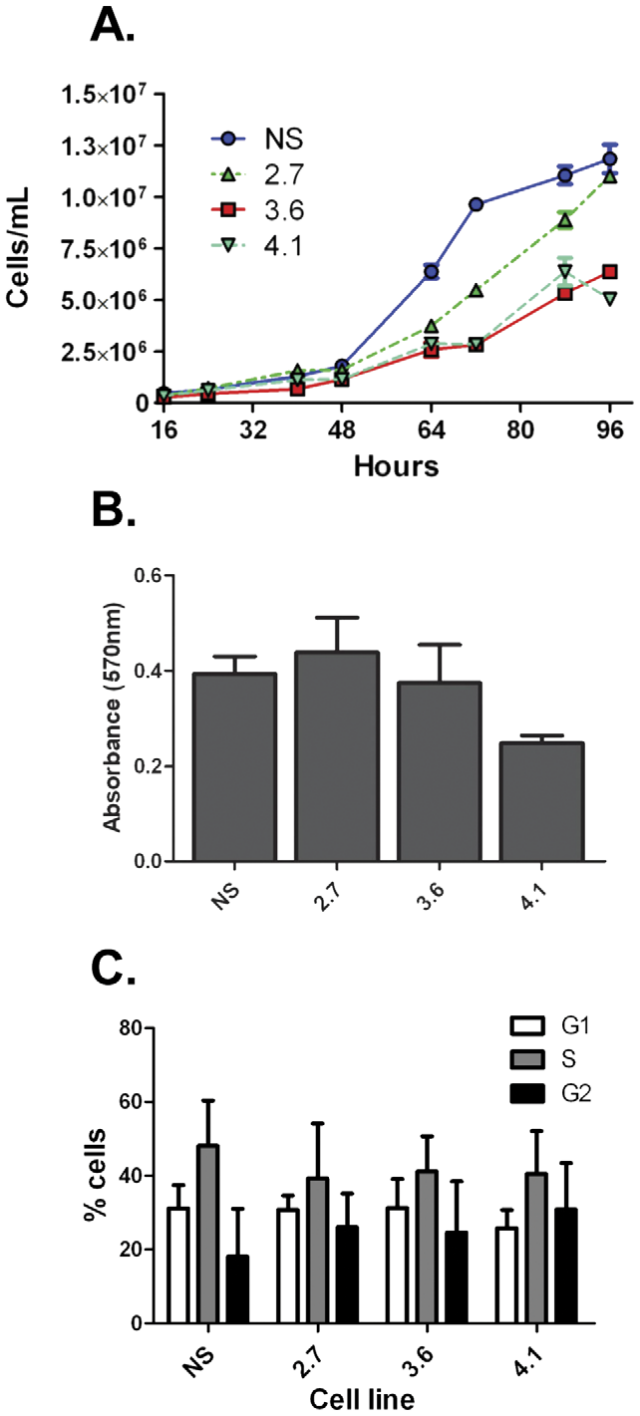
Characterization of shLRPPRC cells. (**A**) Growth of shLRPPRC cells. NS and shLRPPRC cells were seeded in triplicate and counted with a cell counter every 8 and 24 h for a total of 96 h. Data shown is representative of two experiments and error bars denote SD. (**B**) MTT assay of NS and shLRPPRC cells. Cells were seeded in a 96-well plate and were incubated for 24 h at 37°C prior to addition of the dye reagent. Cells were incubated an additional 2 h and absorbance measured. Assay was performed in triplicate and data is representative of three independent experiments. Error bars denote SD. (**C**) Cell cycle profiles of NS and shLRPPRC cells. Proliferating cells were stained with Vindelov's reagent and analyzed by flow cytometry to determine the percent of cells in each phase of the cell cycle. Data shown is representative of three independent experiments and the error bars denote SD.

LRPPRC reportedly modulates cellular proliferation by regulating the expression of factors necessary for the progression of the cell cycle and proper mitochondrial function. Moreover, HIV-1 infection is known to modulate metabolic pathways during productive replication [67], [68]. Combined these findings suggested a possible mechanism for the observed effect of LRPPRC depletion on HIV-1 infectivity. To determine if the LRPPRC-depleted cells had an altered proliferation phenotype, we measured the growth rate of each shLRPPRC cell line. Cell lines were seeded at low confluency and growth rate monitored by counting cells at 8 or 16 h intervals for 96 h. Two of the three LRPPRC depleted cell lines, 3.6 and 4.1, displayed a ≥2-fold reduction in the proliferation rate compared to the 293T.NS control cells (Fig. 6A). Indeed, the growth curves correlated with the overall level of LRPPRC knockdown and the expression of nuclear LRPPRC (Fig. 2). The partially depleted 2.7 cell line that retained nuclear LRPPRC grew slightly slower than the NS cells, whereas both the 3.6 and 4.1 cells grew at a rate approximately half that of the NS cells.

Since LRPPRC is implicated in the regulation of mitochondrial metabolism the mitochondrial health of each cell line was evaluated. We first checked general mitochondrial enzymatic activity by MTT assay. Both the 2.7 and 3.6 cell lines produced comparable levels of MTT as the NS cells (Fig. 6B). There was a slight reduction in MTT activity in the 4.1 cell line, but it was ≤2-fold compared to the NS cell line. Second, we measured the mitochondrial membrane integrity as a marker for apoptosis in each cell line by JC-1 dye staining assay; but there were no differences among the cell lines (data not shown). These data suggested that there was no substantial mitochondrial defect in the LRPPRC-depleted cells.

Finally, the cell cycle profiles of the cell lines were determined to detect any defects in cell cycle progression for each cell line. Cells were stained with Vindelov's reagent [69] and cell cycle profiles determined by flow cytometry analysis (Fig. 6C). Overall there was no significant difference between the three shLRPPRC cell lines and the NS cells. The profile of the 2.7 cells was similar to the 293T.NS cells, and the 3.6 and 4.1 cell lines exhibited only a slight increase in cells in the G2 phase complemented by a slight decrease in cells in S phase compared to the 293T.NS cells. The lack of a substantial effect of LRPPRC knockdown on cell cycle profile suggested that cell cycle arrest was not the reason for delayed growth of shLRPPRC cells. Together, the analyses of the shLRPPRC cell lines indicated that the depletion of nuclear LRPPRC resulted in a decrease in cellular proliferation without a substantial reduction or abnormality in cell cycle phenotype.

## Discussion

Retroviral replication is a complex interaction between virus and host factors. These studies identified a new cellular protein, LRPPRC, which is necessary for efficient HIV-1 infection. LRPPRC was identified by mass spectrometry of IN- and MA- protein complexes at 4 hpi, using a biotin-labeling system [38]. LRPPRC, a nucleic acid binding protein [57], [58], [59], was subsequently determined to interact with HIV-1 RNA and DNA during the early steps of replication. Stable knockdown of LRPPRC with three independent shRNAs reduced HIV-1 infection during the early phase of virus replication. Infection of all three cell lines resulted in a reduction of PIC formation, and viral DNA nuclear import was impaired in two of the cell lines. LRPPRC expression was not required for efficient virus production or viral RNA encapsidation. Combined these data indicated that LRPPRC depletion affects the early events of HIV-1 infection by at least two mechanisms.

LRPPRC is a multifunctional protein involved in mitochondrial gene expression and function, cell cycle progression, and RNA regulation. Therefore it was not surprising that knockdown of LRPPRC affected HIV in multiple ways. LRPPRC was detected in the membrane/organelle, nuclear, and insoluble/cytoskeletal fractions, but not the cytosol of cells. Surprisingly, there were distinct patterns of LRPPRC expression and its associated factors in subcellular compartments among the three different shLRPPRC cell lines. In all three cell lines LRPPRC was reduced in the insoluble/cyoskeletal fraction, and there was a loss of membrane/organelle associated SLIRP. The 3.6 and 4.1 cell lines showed a reduction of LRPPRC in the membrane/organelle fraction and nuclei, a loss of nuclear eIF4E, and a loss of c-myc. The 2.7 cells exhibited an only a slight reduction of LRPPRC in the membrane/organelle fraction, but showed no change in the expression of eIF4E or c-Myc. Thus, the 2.7 cell line has a unique knockdown phenotype when compared to the 3.6 and 4.1 cell lines.

The only defect of HIV replication identified in all shLRPPRC cell lines was a loss of PIC formation. Hence, the loss of cytoskeletal-associated LRPPRC correlates with a loss of PIC formation and represents one mechanism affecting HIV-1 replication. Sequence analysis of LRPPRC identified a putative SEC1 domain involved in cytoskeletal interaction [70], however the function of cytoskeletal LRPPRC remains unknown. Disruption of the cytoskeleton reduces RTC function [71] and possibly PIC stability through association with microtubules [72]. Delineation of the role of LRPPRC in this subcellular fraction will provide insight into the mechanism of PIC inhibition.

Infection of the 3.6 and 4.1 cell lines resulted in an impairment in the accumulation of 2-LTR circles in the nuclei of cells, implying that nuclear LRPPRC is necessary for efficient nuclear import of viral DNA. Nuclear LRPPRC regulates the expression of mRNAs related to cell cycle progression through an interaction with eIF4E [63]. eIF4E is a critical regulator of the mRNA export of several cell cycle regulatory factors including Cyclin D1, Pim-1, and c-Myc [63]. Knockdown of LRPPRC adversely affects eIF4E mediated RNA export due to cytoplasmic retention of eIF4E and accumulation in PML-bodies (P-bodies) [63]. Consistent with this study, the level of nuclear eIF4E protein was reduced in the 3.6 and 4.1 cell lines, but not the 2.7 cells. Moreover, the 3.6 and 4.1 cell lines grew at a slower rate and exhibited a substantial reduction in the proliferation factor c-Myc. The loss of nuclear eIF4E and the strong reduction in c-Myc levels in these cell lines likely contributed to the loss of proliferation compared to the NS or 2.7 cell lines. Consistent with previous studies, MLV infectivity, which is dependent on cell division, was reduced only in the 3.6 and 4.1 cell lines. HIV-1 infection is not prohibited in growth-arrested cells. Therefore the loss of nuclear LRPPRC resulted in a loss of viral DNA nuclear import via a distinct mechanism. Additional studies will be needed to determine whether it is the loss of LRPPRC or its associated factors that hinder the early steps of HIV infection by investigating the loss of eIF4E and c-Myc independently.

The other ascribed functions of LRPPRC provide additional means through which its depletion could impact HIV-1 infection. LRPPRC interacts with both RNA and DNA [57], [58], [59]. HIV replication is regulated extensively at the RNA level and numerous studies implicated RNA processing factors such as the serine/arginine-rich family of proteins as critical for HIV-1 infection [73], [74]. These proteins are vital for mRNA nuclear export, nonsense-mediated decay, and mRNA translation. Moreover, the perturbation of eIF4E, a critical regulator of other transcription or translation factors including Cyclin D1, and Pim-1, may also result in an inhibition of HIV-1 infection.

Finally, the loss of mitochondrial LRPPRC may also contribute to the reduction of HIV-1 infection. The majority of the data suggests that LRPPRC is primarily a mitochondrial associated protein [57], [62], [64] and new evidence confirm that it is required for translation of mitochondrial mRNAs [75]. In addition to proper mitochondrial function and gene expression [61], LRPPRC is essential for COX I and III mRNA expression [62], and phosphoenolpyruvate carboxykinase and glucose-6-phosphate expression [60]. LRPPRC-depleted MCH58 fibroblast cells exhibit decreased expression of three mitochondrial gene sets: O-glycan biosynthesis, glycosphingolipid biosynthesis, and glycosphingolipid metabolism [76]. The effect of perturbing mitochondrial function on HIV-1 infection is not known, but it is clear that virus infection alters mitochondrial function. Two independent proteomic analyses observed the up-regulated expression of proteins associated with mitochondria, the TCA cycle, fatty acid oxidation, and oxidative phosphorylation in response to HIV-1 infection [67], [77]. Thus LRPPRC reduction could negatively impact HIV-1 infection by altering mitochondrial health and reducing the function of critical metabolic pathways to a level that does not support efficient virus replication.

A significant reduction of mitochondrial LRPPRC was observed in the 3.6 and 4.1 shRNA cell lines. Despite this, there was no measurable disruption of mitochondrial function in the cells as measured by MTT and JC-1 assays. These results indicate that there was no correlation between the loss of infection and mitochondrial function; however they do not exclude the possibility that mitochondrial associated LRPPRC is critical for HIV-1 infection. Over extended cell passages LRPPRC accumulated in the mitochondrial compartment in both the 3.6 and 4.1 cell lines (data not shown). This suggests that mitochondrial LRPPRC may have a long half-life in this compartment. Moreover, there was a reduction of the LRPPRC-associated protein SLIRP in all three cell lines. SLIRP is important for posttranscriptional mitochondrial mRNA expression and consequently oxidative phosphorylation [64], [65]. Interestingly, this primarily mitochondrial protein also binds the steroid receptor RNA activator which is an RNA species that acts as a nuclear receptor coactivator [78]. As with eIF4E and c-Myc, individual knockdown of SLIRP will need to be investigated to determine what role it may play in HIV replication. Future examination of SLIRP and additional metabolic indicators and/or a gene array analysis of the shLRPPRC cells may reveal new pathways that are critical for efficient HIV infection.

## Supporting Information

Figure S1 **Separation of biotinylated IN and MA protein complexes.** Following affinity purification, SA-agarose bound protein complexes were separated by SDS-PAGE. The gel was stained by SYPRO Ruby and imaged at 450 nm. Arrows denote bands representing IN and MA. MW markers are shown on the left side of the gel. Gel is representative of one of six replicates.
(TIF)

Table S1 List of all unique proteins identified from the mass spectrometry analysis. Column A lists the gene name found in the NCBI database. Column B lists the total number of protein hits from all replicates and columns C & D provide the number of hits in either pull-down (IN or MA). The final three columns provide the total peptide hits, the total Mascot score, and the mean Mascot score for each protein, respectively. The proteins were sorted by mean Mascot score largest to smallest.
(XLSX)

Table S2 List of HIV-1 proteins identified in the MS analysis.
(DOCX)
