# Supplementary figures and images for: Knockdown of the Cellular Protein LRPPRC Attenuates HIV-1 Infection
Source: PLoS One. 2012 Jul 12;7(7):e40537. doi: 10.1371/journal.pone.0040537 (PMC3395635; doi:10.1371/journal.pone.0040537)

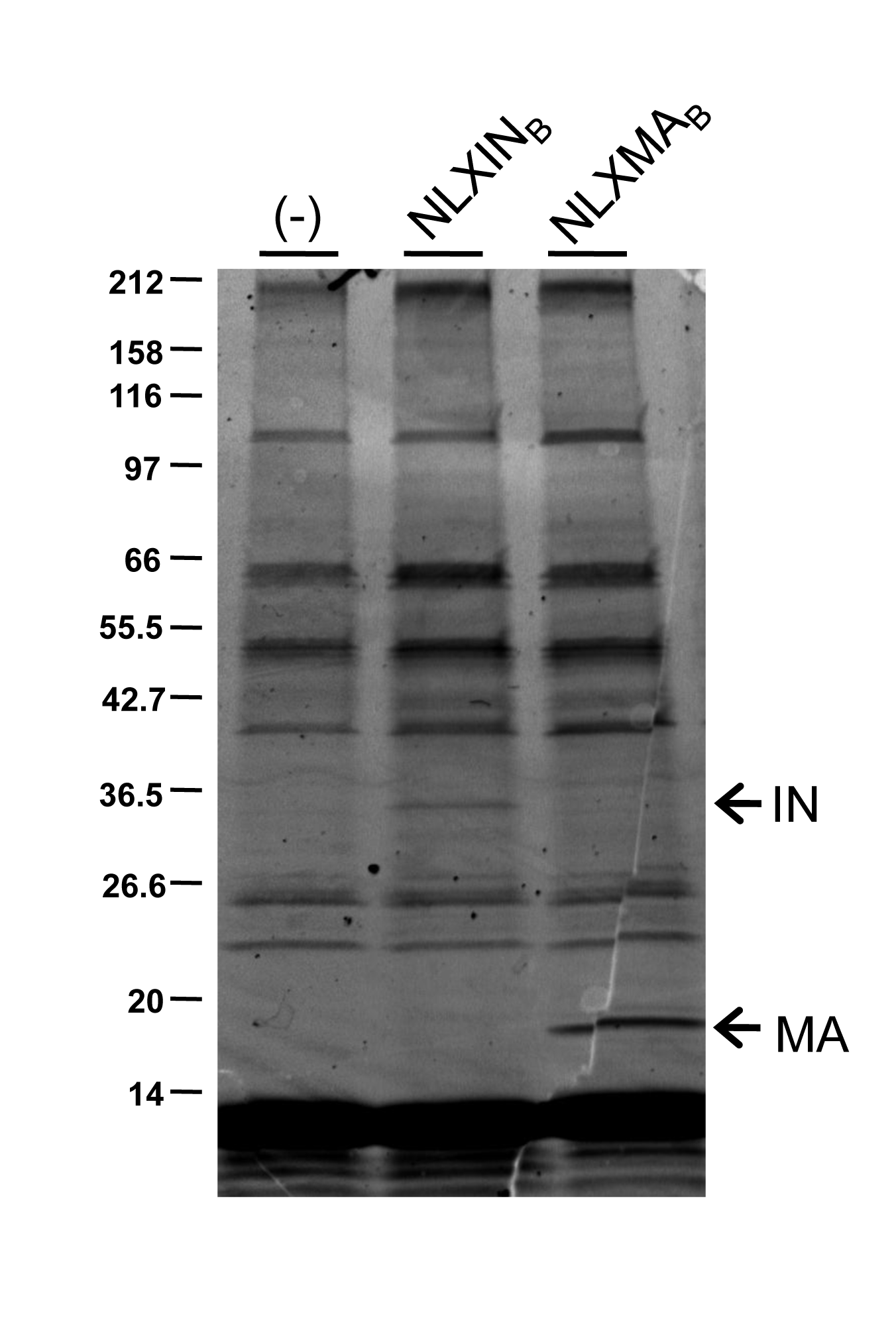

Supplement: Figure S1 — Separation of biotinylated IN and MA protein complexes. Following affinity purification, SA-agarose bound protein complexes were separated by SDS-PAGE. The gel was stained by SYPRO Ruby and imaged at 450 nm. Arrows denote bands representing IN and MA. MW markers are shown on the left side of the gel. Gel is representative of one of six replicates. (TIF) [file pone.0040537.s001.tif]
