# Supplementary material for: Knockdown of the Cellular Protein LRPPRC Attenuates HIV-1 Infection
Source: PLoS One. 2012 Jul 12;7(7):e40537. doi: 10.1371/journal.pone.0040537 (PMC3395635; doi:10.1371/journal.pone.0040537)
Supplement: Table S2 — List of HIV-1 proteins identified in the MS analysis. (DOCX) [file pone.0040537.s003.docx]

| Identification | Peptide Hits | Total Mascot Score | Avg. Score |  | Peptide Hits | Mascot Score | Avg. Score |
| --- | --- | --- | --- | --- | --- | --- | --- |
| Gag | 496 | 25054 | 117.6 |  | 75 | 5137 | 98.8 |
| Pol | 164 | 9124 | 114.1 |  | 388 | 22260 | 127.9 |
| Gag-pol | 24 | 1339 | 121.7 |  | 79 | 4500 | 109.8 |
| Matrix | 64 | 2646 | 105.8 |  | 7 | 342 | 57.0 |
| Protease | 30 | 1839 | 131.4 |  | 46 | 2235 | 101.6 |
| Reverse transcriptase | 6 | 122 | 40.7 |  | 4 | 92 | 127.9 |

| **Matrix IP** | **Integrase IP** |
| --- | --- |

**Table S2. HIV-1 proteins identified in the infected samples by MS analysis**
